# The knowledge and practice towards COVID-19 pandemic prevention among residents of Ethiopia. An online cross-sectional study

**DOI:** 10.1371/journal.pone.0234585

**Published:** 2021-01-28

**Authors:** Daniel Bekele, Tadesse Tolossa, Reta Tsegaye, Wondesen Teshome

**Affiliations:** 1 Department of Statistics, College of Natural and Computational Sciences, Dire Dawa University, Dire Dawa, Ethiopia; 2 Department of Public Health, Institute of Health Science, Wollega University, Nekemte, Ethiopia; 3 Department of Nursing, Institute of Health Sciences, Wollega University, Nekemte, Ethiopia; 1. IRCCS Neuromed 2. Doctors with Africa CUAMM, ITALY

## Abstract

**Background:**

The disease from the novel coronavirus (COVID-19) has been considered as an international concern and a pandemic starting from the declaration of the World Health Organization (WHO) as an outbreak disease.

**Objective:**

The objective of this study is to assess the prevention of knowledge and practices towards the COVID-19 pandemic among the residents of Ethiopia.

**Methods:**

An online cross-sectional study was conducted among a sample of Ethiopian residents via social platforms of the author’s network with popular social media such as Facebook, Telegram, and email. The snowball sampling was employed to recruit participants. In doing so, we collected the responses of 341 participants successfully from April 15 to 22, 2020. The collected data were analyzed by STATA version 14 software and descriptive statistics were employed to summarize the knowledge and practices of the community towards the COVID-19 pandemic.

**Results:**

The majority of respondents 80.5% were male. About 91.2% of the participants heard about the COVID-19 pandemic. Moreover, out of 341 participants 90.0%, 93.8% of them knew that the COVID-19 pandemic was prevented by maintaining social distance and frequent handwashing, respectively. This shows that the prevention knowledge of the participants towards the COVID-19 by maintaining social distance and frequent handwashing was high. However, out of 341 participants only 61%,84% of them practiced social distance and frequent handwashing toward COVID-19, respectively.

**Conclusions:**

The majority of the participants knew the ways to protect themselves from the novel coronavirus (COVID-19), but there was a great problem of changing this prevention knowledge to practices. This shows that there is an action gap between having prevention knowledge of the COVID-19 and implementing it into practices to tackle the spread of the COVID-19 among communities. Therefore, the concerned body should be focused on providing awareness and education for the community regarding the implementation of prevention knowledge to practices.

## Introduction

Coronavirus is one of the major pathogens that mainly targets the respiratory system of the human organ. Previous outbreaks of coronaviruses have been recorded in history as a severe acute respiratory syndrome (SARS)-CoV and the Middle East respiratory syndrome (MERS)-CoV [1]. The new coronavirus has been identified as the cause of acute respiratory disease since the end of December 2019. Later, it was labeled as SARS-CoV2 by the World Health Organization as a different strain of coronavirus from SARS and MERS coronaviruses. Here, the difference between them is the genetic make-up, clinical presentations, case fatality, and the rate of spread across the world. That is the SARS-CoV2 is the virus that has caused coronavirus disease 2019 (COVID-19) became the newest virus causing global health fear [2, 3].

The COVID-19 symptoms can range from mild (or no symptoms) to severe illness and are mainly characterized by fever, dry cough, dyspnea, headache, sore throat, and rhinorrhea, and sometimes hemoptysis [4, 5]. The main route of transmission is close contact (about 6 feet or two arm lengths) with a person who has COVID-19 respiratory droplets when an infected person coughs, sneezes, or talks and touching a surface or object that has the virus on it, and then touching his/her mouth, nose, or eyes [6].

Currently, more than 2.2 million people are infected and more than 152 thousand die of coronaviruses globally as of 19 April 2020 [7]. Moreover, this figure is still increasing rapidly from time to time globally. For instance, in the case of Africa, almost all countries have now confirmed the cases and the number of deaths is increasing rapidly. Thus, if the spread of the disease is not well managed on time, it will have an impact on the African economy extensively. This is because Africa’s fragile health systems, coupled with a high burden of COVID-19 would cost the continent. The speed at which countries can detect reports and respond to outbreaks can reflect their wider institutional capacity [8, 9]. The threat of COVID-19 to health systems in Africa can be compared with a metaphor that says ‘the eye of the crocodile’ which means in the lake, only the eyes of the crocodile are visible on the surface while the rest of the body is immersed in water. In this viewpoint, the eyes represent public health preparedness, while the body of the crocodile represents Africa’s fragile health systems [10].

According to the Ministry of Health, in Ethiopia, the number of infected cases reached 117 and 3 deaths as of 24 April 2020. In a developing country like Ethiopia, where trained human resources and equipment for the treatment of COVID-19 are scarce, working on prevention of the viral spread should be a prioritized and feasible intervention. The government of Ethiopia has declared a state of emergency to minimize and stop the spread of this evil disease, COVID 19. The state of emergencies includes staying home as much as possible, avoiding close contact with others, cleaning and disinfecting frequently touched surfaces, washing hands often with soap and water for at least 20 seconds, or using hand sanitizers containing at least 60% alcohol [4].

In Africa, many people have the misconception that they may acquire infection if they visit a healthcare facility due to a lack of trust in the care provided by healthcare facilities and thus, often take preventive and care measures on their own. According to a health behavior model, information knowledge and practice have been identified as important factors for increasing hospital visits among patients with low levels of trust [11].

The Ethiopian government has been very active in disseminating prevention messages on radio and television, at federal and regional levels and there are strong initiatives and recognition of the public health importance of COVID-19 (screening, quarantine, and treatment centers). However, there is still a strong need to enhance community awareness and practices to stop the nationwide spread of the virus [12, 13].

Successful mitigation and reduction of mortality and morbidity due to COVID-19 require behavioral change, which is influenced by people’s knowledge and perceptions [14]. However, currently, the knowledge and practice level of the public towards Covid-19 has not been studied yet. Thus, this study is intended to identify the level of knowledge and practice towards Covid-19 among residents in Ethiopia.

## Methods and materials

This study employed an online cross-sectional survey using Facebook, Telegram, and email to collect data from the respondents regarding their prevention knowledge and practice towards the COVID-19 pandemic from April 15 to 22, 2020. Since the first cases of the COVID-19 were confirmed in Ethiopia on March 13, 2020, the government declared a state of emergency and lockdown practical interaction between the people of Ethiopia on April 5, 2020. This imposed a great problem on researchers to conduct community or institutional-based surveys.

Thus, the researchers selected respondents from the population of Ethiopia by using a snowball sampling technique through the author’s network with residents on the popular social media, Facebook, Telegram, and email. The link to the questionnaire was posted by the author on the above-mentioned social media to be fulfilled. The Terms and Conditions of the website regarding the responses of respondents, the following questions were added at the end of the questionnaire on Google Forms to help the respondents to submit their responses if they agreed with the Terms and Conditions of the Google Forms: “By submitting this form, do you agree to the Terms and Conditions of Google Forms?” In doing so, we were too confidential that our data collection method was compiled with the Terms and Conditions of the Google Form website.

The questionnaire contained both open-ended and closed-ended questions that focused on the respondent’s socio-demographic, knowledge and practice towards COVID-19 prevention. The inclusion criteria of the participants used in this study were the ability of the respondents to read, write, and understand the English language, having Ethiopian nationality, and aged ≥ 18 years old. Before proceeding to fulfill the questionnaire, the objective of the study, the informed consent of participation, the declaration of anonymity and confidentiality of their responses was briefed in a good manner in the questionnaire [7].

### Measurements and data management

The knowledge and practice towards COVID-19 prevention were measured based on the WHO (2020) Survey Tool and Guidance [15]. This study employed descriptive statistics to summarize the knowledge and practice of their respondents towards novel coronavirus pandemic prevention [16]. The questions about the knowledge of COVID 19 prevention had 7 items as presented in Table 2 below, and the questions about the respondent’s practice towards COVID 19 prevention had 6 items, as presented in Table 3. The rest of the five questions were about the respondent’s socio-demographic information. All the questions contained the category of (“Yes”, “No”, and “Don’t know”). Prevention knowledge and practice level were assessed by assigning one point for each correct answer and the knowledge towards COVID 19 prevention indicated by two categories: poor for (< 5 of 7 items) and good for (≥ 5 of 7 items). Especially regarding the towards COVID-19, the respondents were asked about going to crowded places, wearing masks in public, maintaining social distance, handwashing, avoiding handshaking, and obeying government restrictions. The respondents practiced towards COVID-19 indicated by two categories: poor practice for (< 4 of 6 items) and good practice for (≥ 4 of 6 items).

The collected data were checked manually for its completeness. The data were coded and edited in Epi-Info version 3.5.1, exported to STATA version 14.0, for further statistical data analysis. Finally, descriptive statistics were employed to summarize the data.

### Ethical approval and considerations

The study was conducted by preparing an online survey. Thus, the investigators informed the study participants about the general objective of the research that their answers remained anonymous to ensure the confidentiality of the information they provided. Therefore, every respondent’s consent for participation was informed before proceeding to fill the questionnaire. Moreover, the questionnaire was designed anonymously and the study result did not identify the personality of the respondents but presented in aggregated statistics.

## Results

### Socio-demographic characteristics of the respondents

A total of 341 respondents participated in this study. The majority of the respondents (80.3%) were male. Among those participants, more than two-thirds of them were in the age group of 18–32 years old and had an education level of college diploma and above. Among all respondents, more than half of them were unmarried, about two-thirds of them were urban residents. (Table 1) presents details.

**Table 1:** Sociodemographic characteristics of respondents to assess prevention knowledge and practice among the community towards the COVID-19 pandemic in Ethiopia (N = 341).

Participant characteristics	Levels	Number	Percent (%)
Gender	Male	274	80.3
	Female	67	19.7
Age	18–32	233	68.3
	33–46	108	31.7
Marital status	Single	197	57.8
	Married	144	42.2
Level of education	Secondary	101	29.6
	Higher	240	70.4
Residence	Urban	227	66.6
	Suburban	59	17.3
	Rural	55	16.1

### Knowledge of respondents towards COVID-19 prevention

In the current study, from all respondents, the majority (91.8%) had information about COVID-19. The analysis showed that although the majority of the respondents thought to avoid touching the nose, eye, and face with an unwashed hand to protect themselves from getting COVID-19, about 11.4% of the respondents did not agree with this preventive measure. Besides, 286 (83.9%) out of all the respondents thought that wearing the mask could protect them from getting off the COVID-19, and the rest of 16.1% of the respondents did not believe that wearing a face mask could protect from getting off the COVID-19. Furthermore, the majority of the respondents are three-fourths, reported that avoiding hugging with people can protect them from getting the infection of COVID-19. Also, 26.7% of all respondents thought that drinking a lot of water can protect them from getting an infection of the COVID-19. However, more than 93% of the respondents reported that maintaining social distance can protect them from getting off the COVID-19, and 90% of them thought that frequent hand washing for 20 seconds can protect them from getting an infection with the virus. (Table 2) presents details.

**Table 2:** Frequency distribution of respondents’ knowledge of prevention from Covid-19, Ethiopia (N = 341).

Knowledge related questions on COVID-19	Yes	No
	N (%)	N (%)
Have you heard of COVID-19?	313(91.8)	28(8.2)
Do you think to avoid touching nose, eye, and face with unwashed hand protect COVID-19?	302(88.6)	39(11.4)
Do you think wearing a mask protects you from COVID-19?	286(83.9)	55(16.1)
Do you think to avoid hugging with people protect from COVID-19?	263(77.1)	78(22.9)
Do you think drinking a lot of water can protect you from COVID-19?	91(26.7)	250(73.3)
Do you think maintaining social distance can protect you from COVID-19?	320(93.8)	21(6.2)
Do you think frequently washing hands for 20 seconds can protect COVID-19?	307(90.0)	34(10.0)

**Fig 1:**
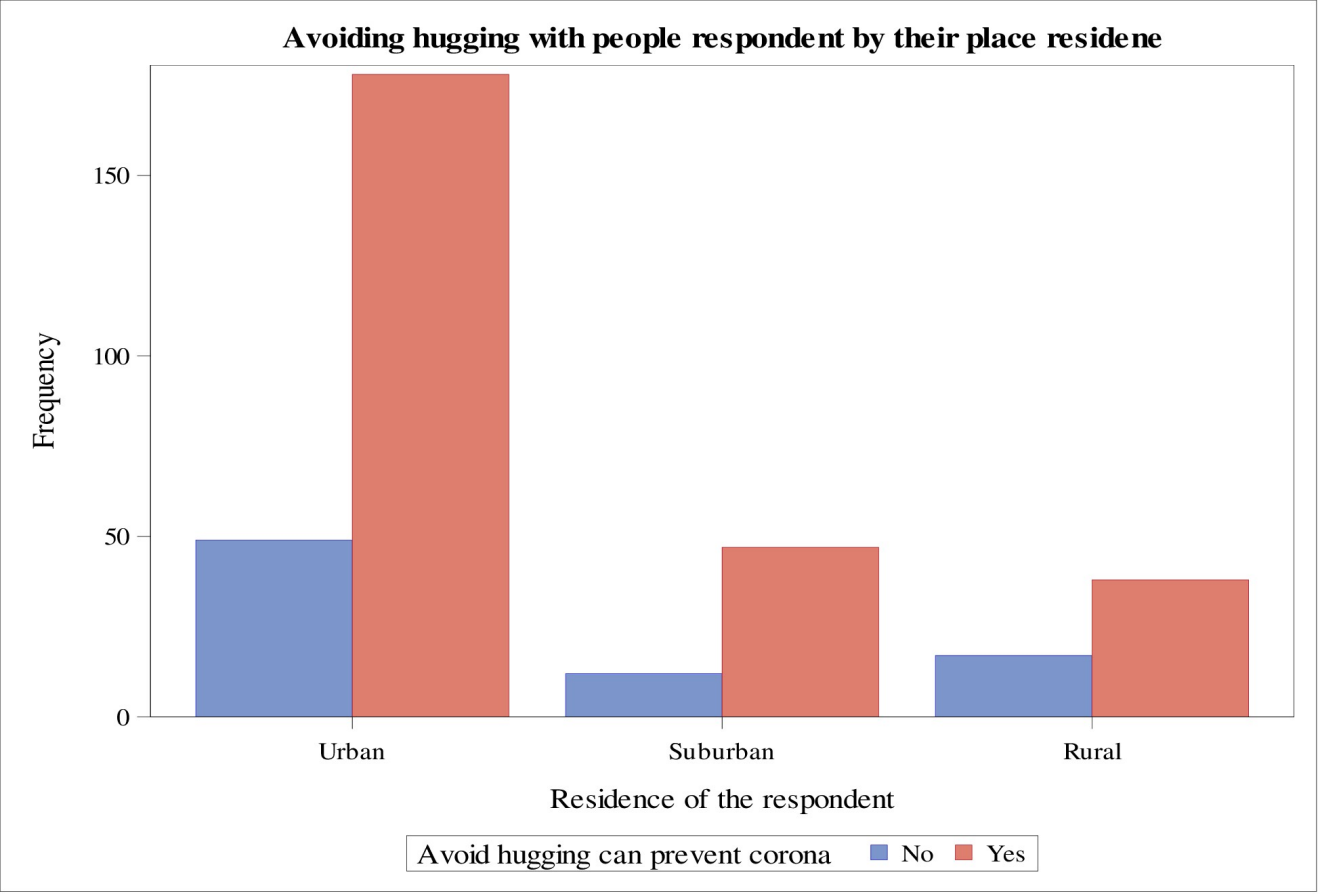
Respondents’ knowledge on avoiding hugging people for the protection of COVID-19 by their place of residence, Ethiopia, May 2020.

**Fig 2:**
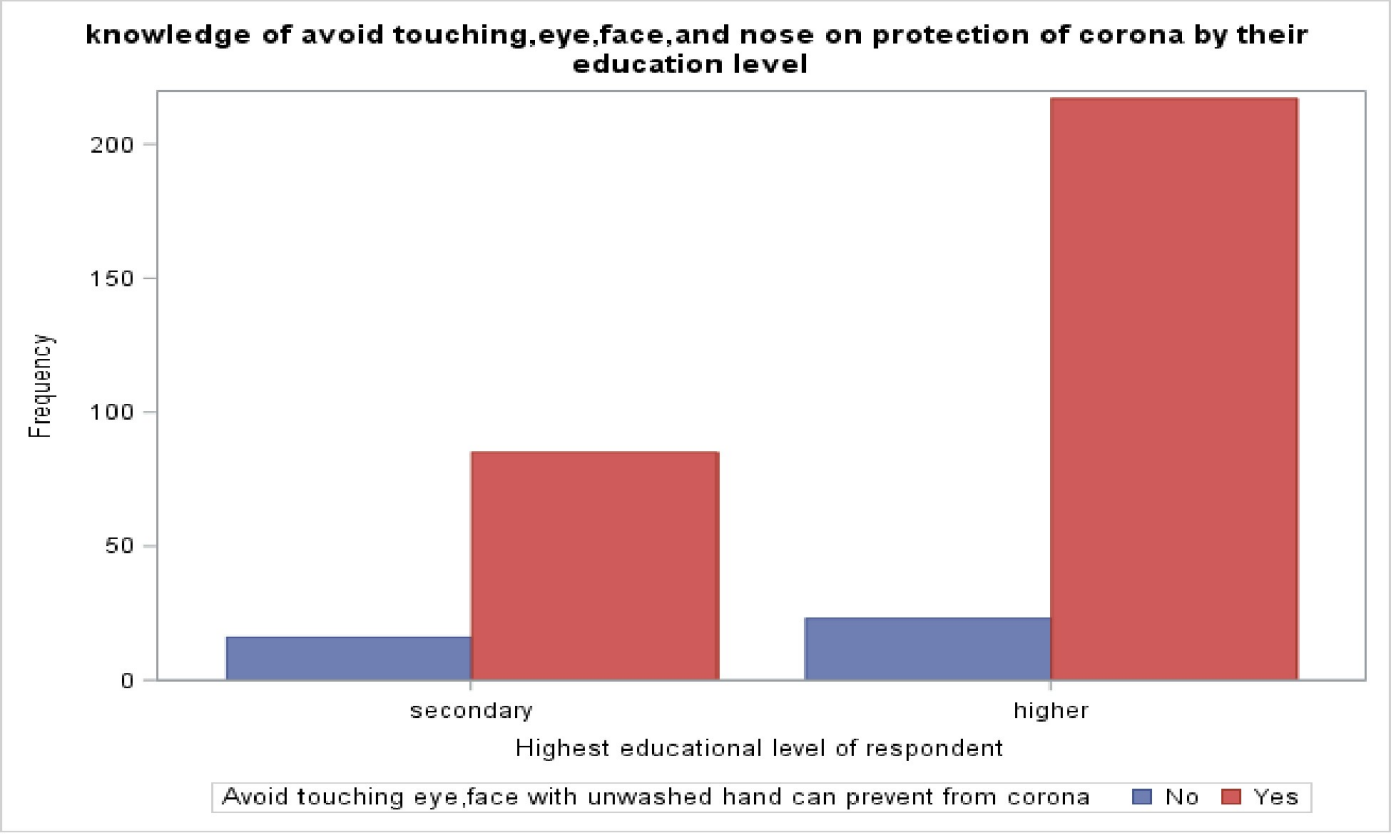
Respondents’ knowledge on avoiding touching eye, nose, and face for the protection of COVID-19 by their educational level, Ethiopia, May 2020.

**Fig 3:**
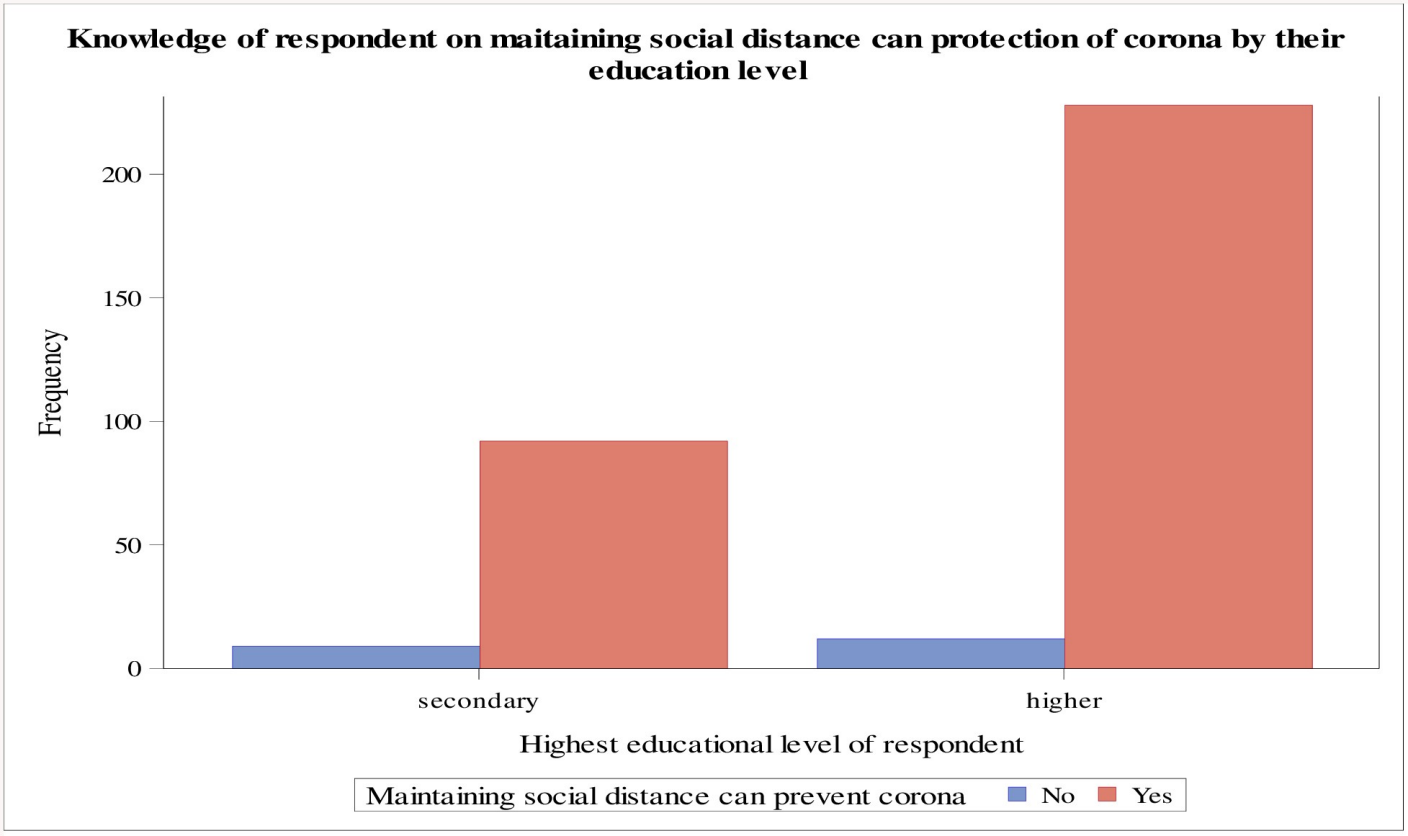
Respondents’ knowledge on maintaining social distance for the protection of COVID-19 by their educational level, Ethiopia, May 2020.

The data revealed that urban area residents have a good knowledge of COVID-19 prevention by avoiding hugging with people than the respondents living in suburban and rural areas, respectively (Fig 1). Moreover, the majority of the respondents who had the highest educational level knew that avoiding touching the eye, face, and nose with the unwashed hand could reduce the risk of becoming infected with the novel coronavirus (COVID-19) (Fig 2). Also, the majority of the respondents who had the highest educational level reported that maintaining social distance can reduce the risk of getting a novel coronavirus (COVID-19). On the other hand, respondents with a secondary level of education and below had less knowledge on maintaining social distance to reduce the risk of getting the COVID-19 (Fig 3).

### The practice of respondents toward COVID-19 prevention

In this study, it was observed that about 77.4% of the respondents were not obeying government restrictions; however, only about 22.6% of the respondents were obeying government restrictions on COVID-19 prevention. Moreover, 137(40.2%) of the respondents were still going to crowded places, and 76% of them did not wear face masks when they left their homes. The data also revealed that 61.1% of the respondents kept themselves two meters away from each other in a public place. On the contrary, 39.4% of the respondents did not experience social distance in the public place to save themselves from the transmission of novel coronaviruses. Moreover, the data show that the majority (84.5%) of the respondents washed their hands when they went to the public place, and 20.8% of the respondents were still shaking their hands with people when they went to the public place. (Table 3) presents details.

**Table 3:** Frequency distribution of respondents response on their practice towards Novel Coronavirus (COVID-19) pandemic in Ethiopia (N = 341).

Practice related questions towards COVID-19	Yes	No
	N (%)	N (%)
Are you obeying government restrictions on COVID-19 prevention strategies?	264(77.4)	77(22.6)
Are you going to a public or crowded place after the COVID-19 pandemic confirmed?	137(40.2)	204(59.8)
Did you wear a mask when you leave your home?	82(24.0)	259(76.0)
Did you keep yourself two meters from another person when you go to the public?	210(61.6)	131(38.4)
Did you make a handshake with a person when you go to the public?	71(20.8)	270(79.2)
Did you wash your hand at the place you went to or in the public?	288(84.5)	53(15.5)

**Fig 4:**
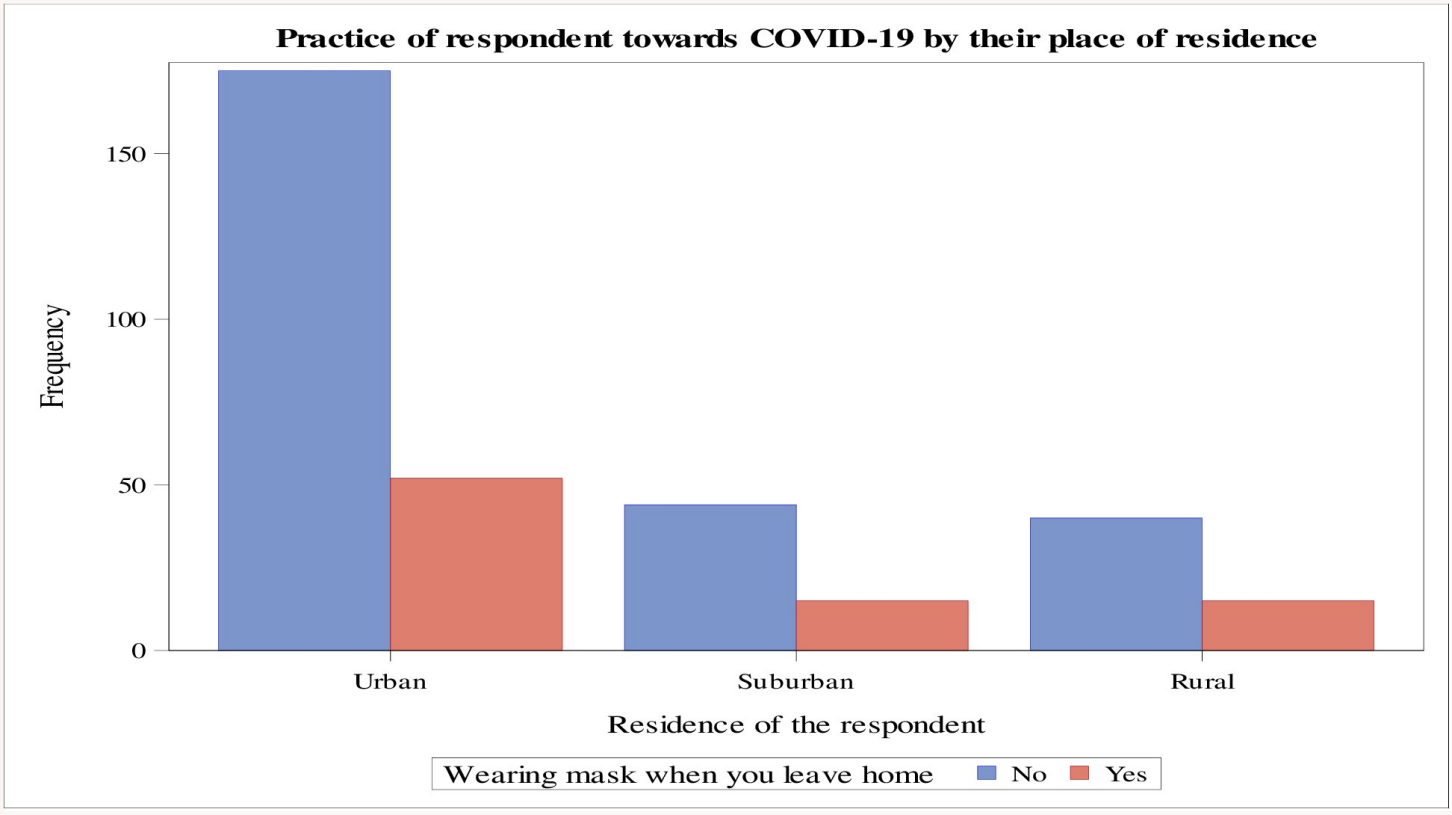
Respondents’ practice of wearing masks in public places by their place of residence, Ethiopia, May 2020.

**Fig 5:**
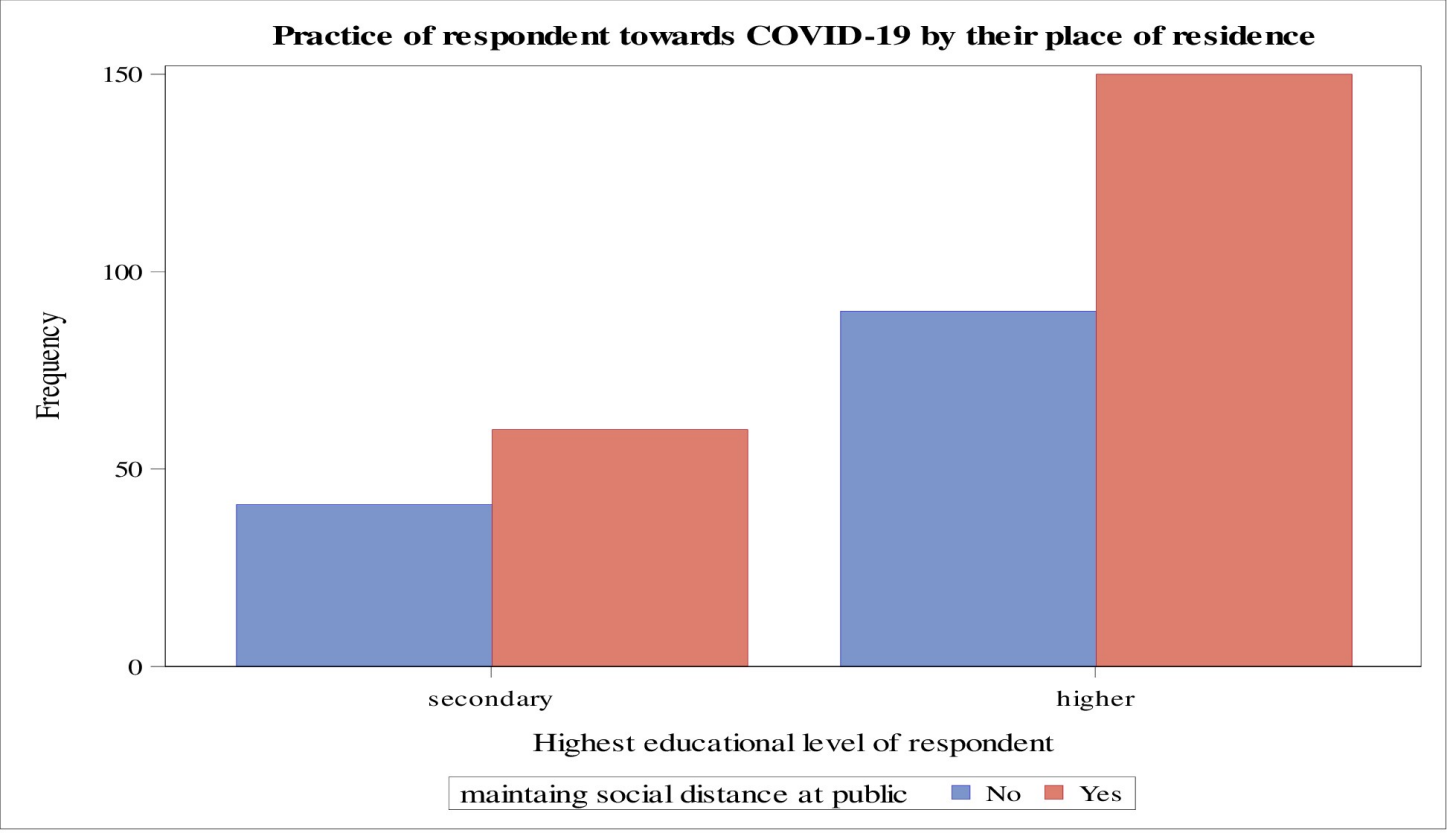
Respondents’ practice of maintaining social distance by their educational level, Ethiopia, May 2020.

The majority of respondents living in urban areas have a good implementation of prevention knowledge of the COVID-19 pandemic when wearing a face mask when they went to the public place as compared to the respondents living in suburban and rural areas (Fig 4). Furthermore, those respondents with the highest educational level have a good practice of prevention knowledge of the COVID-19 pandemic by maintaining the social distance (i.e., at least keeping themselves two meters away from another person) as compared to the respondents with secondary school educational level (Fig 5).

Generally, the mean score and standard deviation of the respondent’s prevention knowledge of the COVID-19 pandemic was 5.5 ± 1.1 or the proportion of respondents know COVID-19 prevention techniques was 78.6%, and the mean score and standard deviation of the respondents on the practice of prevention knowledge of the COVID-19 pandemic was 3.1 ± 1.1 or the proportion of respondents who practiced COVID-19 prevention was 51.5%. (Table 4) presents details.

**Table 4:** The knowledge and practice score of the respondents towards COVID-19 pandemic prevention (N = 341).

Variables	Mean ± SD	Score range	Percent
Knowledge score on prevention COVID-19	5.5 ± 1.1	Range (0–7)	78.9%
Practice score on prevention of COVID-19	3.1 ± 1.1	Range (0–6)	51.5%

The Chi-square test of associations shows that age, gender, marital status, educational level, and place of residence are categorical variables independents variables associated with knowledge and practice towards COVID-19 of the respondents. (Table 5) presents details.

**Table 5:** The bivariate analysis of the sociodemographic factors with their knowledge and practice towards COVID-19 among residents of Ethiopia (N = 341).

Variable	Knowledge of COVID-19		Chi-square	Practices towards COVID-19		Chi-square
	No	Yes		No	Yes	
Age						
18–32	35(15.0%)	198(85.0%)	<0.001	166(71.2%)	67(28.8%)	<0.001
33–46	17(15.7%)	91(84.3%)		75(69.4%)	33(30.6%)	
Gender						
Male	33(12.0%)	241(88.0%)	<0.001	193(70.4%)	81(29.6%)	<0.001
Female	19(28.4%)	48(71.6%)		48(71.6%)	19(28.4%)	
Marital						
Single	29(14.7%)	168(85.3%)	<0.001	132(67.0%)	65(33.0%)	<0.001
Married	23(16.0%)	121(84.0%)		109(75.7%)	35(24.3%)	
Education						
Secondary	25(24.8%)	76(75.2%)	<0.001	71(70.3%)	30(29.7%)	<0.001
Higher	27(11.2%)	213(88.8%)		170(70.8%)	70(29.2%)	
Residence						
Urban	29(12.8%)	198(87.2%)	<0.001	161(70.9%)	66(29.1%)	<0.001
Suburban	7(11.9%)	52(88.1%)		43(72.9%)	16(27.1%)	
Rural	16(29.1%)	39(70.9%)		37(67.3%)	18(32.7%)	

In this study, the collected data revealed that 241(88.0%) and 81(29.6%) males had knowledge of COVID-19 and practice towards COVID-19, respectively. The 198(85.0%) and 67(28.8%) of the respondents aged 18–32 knew about the COVID-19 prevention and practice towards COVID-19, respectively. (Table 5) presents details.

## Discussion

This study was the original study conducted to assess the knowledge and practice of the COVID- 19 pandemics among the residents of Ethiopia. Accordingly, the prevention knowledge of the respondents towards the COVID-19 was high and the practices towards the COVID-19 pandemic were low among the community.

In the current study, approximately 91.2% of the Ethiopian population had information about novel coronaviruses at the time when the study was conducted. It is comparable with a study conducted in Pakistan in which more than 90% of medical students had heard about the disease [9]. Also, this finding is in line with a study conducted in Nepal, in which 91.6% of the population were aware of all the clinical features of novel coronavirus[17]. A survey conducted in three countries of Africa (Nigeria, South Africa, and Kenya) showed that more than 94% of the population had information about the novel coronavirus [1].

Based on the knowledge score of the participants, the overall prevention knowledge towards the COVID 19 pandemic was 78.8%. This finding is in line with studies conducted in Jordan [18], Nigeria [19], and an online survey conducted in Uganda [20], in which respondents had good knowledge of COVID 19 prevention. However, this finding is lower than that of a study conducted in China [21]. In this country, the knowledge of HCW towards COVID 19 prevention was high (89%). Furthermore, this finding is lower than the study conducted in Pakistan in which the knowledge towards COVID 19 prevention was 92.3% [22]. This discrepancy could be due to differences in the study population. The previous study was conducted among health professionals to assess their knowledge level, but the current study was conducted among the general population. This is because health professionals have more exposure to information about the disease.

This result is higher than the study conducted in Addis Zemen Hospital, North West Ethiopia, in which the knowledge of participants towards COVID 19 was 33.9% [14]. This difference might be due to the difference in study participants, in which the previous study was conducted among chronic disease patients, while the current study was conducted among the general population of the country. Furthermore, the previous study was conducted through face-to-face interviews, while the current study was conducted via an online data collection. Therefore, knowledge among the general population is higher than chronic disease patients because most of the respondents participated in the current study literate population.

The findings of this study were higher than the study conducted in Nigeria among pregnant women. In Nigeria, the result of a study showed that the knowledge of pregnant women regarding the preventive mechanism of COVID 19 was 60.9% [23]. This difference might be seen due to the time of the study and the population participated in the study. In the previous study, the study was conducted when the disease was declared as a pandemic for the first time, while our study was conducted after the population provided with awareness about the disease. Besides, pregnant women were the target population, while all eligible residents have participated in the current study.

In the current study, more than 90% of the population had a good knowledge with regards to maintaining social distance and frequently washing hands for greater than 20 seconds. These are the two main strategies that were recommended by WHO to prevent the transmission of this disease [24].

Even though the knowledge regarding social distancing and frequent hand washing was good, the practice of experiencing social support and frequent hand washing is low when compared to their knowledge level. Furthermore, in this study, only 61% and 84% of participants practice social distancing and hand washing, respectively. Moreover, around 88% of participants have good knowledge that wearing a mask can protect people from COVID 19. Similarly, 82% of participants were practiced to wear a mask to prevent COVID 19 in the Ethiopian context.

Overall, there is a gap between having knowledge and practicing the prevention mechanism of COVID 19. This could be due to financial scarcity to practice prevention mechanisms, trained health professionals on the disease. The scarcity of PPE could be one reason for the observed gap. Due to the increased need for PPE, it was a global problem in combating the pandemic. Moreover, the strong social interdependence challenges the main prevention practices such as social distancing in Ethiopia [25]. On the other hand, continued political insecurity in some regions of the country would also the main reason for not practicing the prevention method of the COVID-19 pandemic. Moreover, the economic status of the population is substandard and their life is depending on daily activities.

### Strengths and limitations of the study

One of the strengths of this study is that it is the study conducted for the first time in Ethiopia to assess the prevention knowledge and practice of prevention knowledge towards the Novel Coronavirus diseases for the Ethiopian community. However, there are some limitations to this research. Among those one of the key limitations is that questions that are related to the knowledge and practices of the Ethiopian community were not validated. The second limitation of this study is that the data collected by an online distribution of the survey through social media and only individuals with access to social media and the internet participated in the study. Thus, the study may not represent the overall population of the country.

## Conclusions

In this study, the knowledge of the respondents towards COVID-19 pandemic prevention was high. However, the implementation of prevention knowledge towards the COVID-19 of the respondents was low as compared to those respondents who knew about the prevention of the COVID-19 pandemic. This shows that changing the prevention knowledge to practices toward the COVID-19 pandemic was poor in the community. Therefore, to tackle the spread of this COVID-19 pandemic disease in the community, prevention knowledge of the community should be implemented into the practices. To do so, the stakeholders should play their vital role to teach the community about the ways and mechanisms of implementing the prevention knowledge into practice to combat the spread of the COVID-19 pandemic diseases. We recommend community-based awareness creation and education to change the prevention knowledge into practices to combat the spread of the COVID-19 pandemic.

## Supporting information

S1 Data The knowledge and practice towards COVID-19 among residents of Ethiopia an online cross-sectional survey questionnaire. (DOCX)
