# Supplementary material for: The knowledge and practice towards COVID-19 pandemic prevention among residents of Ethiopia. An online cross-sectional study
Source: PLoS One. 2021 Jan 28;16(1):e0234585. doi: 10.1371/journal.pone.0234585 (PMC7843015; doi:10.1371/journal.pone.0234585)
Supplement: S1 Data — (DOCX) [file pone.0234585.s001.docx]

Top of Form

**Online survey on COVID-19 pandemic**

Dear respondents;
The purpose of this survey is to assess knowledge and practice toward COVID-19 pandemic disease prevention among Ethiopian residents. This can provide a greater opportunity to understand the existing gaps between COVID-19 awareness and practice among the general public and used to scale up the intervention strategies. Thus, we invite you to participate in this short survey and provide your valuable opinion regarding the COVID-19 pandemic.

Your answer will not be released at anyone and remain anonymous. The result of this study was only presented in aggregated statistics and the individual profile of respondents was not identified. Your participation is voluntary and you may choose to stop the filling of the question at any time.

If you need more inquiry regarding this survey please contact us on [daniadvent12@gmail.com](mailto:daniadvent12@gmail.com) or [yadanotolasa@gmail.com](mailto:yadanotolasa@gmail.com) or [retatg@gmail.com](mailto:retatg@gmail.com)

* Required

How old were you at your last birthday (in years)? *

Your answer

What is your gender? *

Male

Female

What is your current marital status? *

Single

Married

Other (divorced, widowed)

What is your highest educational level you have completed? *

Elementary (1-8th grade)

High school (9-10th grade)

University degree

Preparatory School (11-12th grade)

University Master degree and above

College Diploma

Other:

How do you categorizes place where do you live? *

Urban

Suburban

Rural

Have you heard of the Novel Coronavirus (COVID-19)? *

Yes

No

Don't know

If your answer is "Yes", from which source you have heard the information? (Multiple answers is possible)

Television

Social media (Facebook, telegram....etc)

From the ministry of health website

Family, friends, neighbours and colleagues

Other:

Do you think that avoid touching nose, eyes and face with unwashed hand can protect from getting of COVID-19? *

Yes

No

Don't know

Do you think that wearing a mask can protect from getting of Coronavirus (COVID-19)?

Yes

No

Don't know

Do you think that avoid hugging with people can protect from getting of Coronavirus (COVID-19)? *

Yes

No

Don't know

Do you think that drinking a lot of water can protect from getting of Coronavirus (COVID-19)? *

Yes

No

Don't know

Do you think that staying 2 meters away from another person (maintaining social distance ) can protect from getting of Coronavirus (COVID-19)? *

Yes

No

Don't know

Do you think that frequently washing hand with soap for 20 seconds can protect from getting of Coronavirus (COVID-19)? *

Yes

No

Don't know

Do you think you are obeying the government authority (I.e., ministry of health) restriction in regards to COVID-19 prevention techniques? *

Yes

No

Don't know

Are you going to a crowded place or public after COVID-19 pandemic disease confirmed in Ethiopia? *

Yes

No

Don't know

Did you worn a mask when you leave your home?

Yes

No

Don't know

Did you keep yourself 2 meters from another person (do you maintain your social distance) when you go to public?

Yes

No

Don't know

Did you make a handshake with a person when you go to the public?

Yes

No

Don't know

Did you wash your hand at the place you went to or in the public?

Yes

No

Don't know

By submitting this form, do you agree to the terms and conditions of Google Forms? *

Yes, I agree

No, I don't agree

Submit

Never submit passwords through Google Forms.

Bottom of Form

This content is neither created nor endorsed by Google. [Report Abuse](https://docs.google.com/forms/u/0/d/e/1FAIpQLSetCW2ggx2KsnCBRWVW_WUL5gJkSqZJz2IM2wJpNzwWUXeAHA/reportabuse?source=https://docs.google.com/forms/d/e/1FAIpQLSetCW2ggx2KsnCBRWVW_WUL5gJkSqZJz2IM2wJpNzwWUXeAHA/viewform) - [Terms of Service](https://policies.google.com/terms) - [Privacy Policy](https://policies.google.com/privacy)

[Forms](https://www.google.com/forms/about/?utm_source=product&utm_medium=forms_logo&utm_campaign=forms)
